# Multimodal art intervention for flourishing in lonely university students: psychological and exploratory neurophysiological outcomes

**DOI:** 10.3389/fpsyg.2026.1908723

**Published:** 2026-09-29

**Authors:** Wen Zhang, Siyu Tian, Ruikai Yuan

**Affiliations:** 1 School of Medical Humanities, Jinzhou Medical University, Jinzhou, China; 2 School of Foreign Languages, Southeast University, Nanjing, China

**Keywords:** aesthetic emotions, loneliness, multimodal arts, psychological flourishing, randomized controlled trial, university students, wearable EEG

## Abstract

**Introduction:**

Loneliness is highly prevalent among university students and is associated with reduced positive functioning. However, evidence concerning multimodal art interventions for flourishing-related outcomes in this population remains limited. This study examined whether an eight-week, group-based multimodal art intervention could improve psychological flourishing, state positive affect, aesthetic experience, and exploratory wearable electroencephalography (EEG) indices among lonely university students.

**Methods:**

Sixty students with elevated loneliness (20-item UCLA Loneliness Scale score ≥ 44) and below-flourishing mental health, as assessed using the Mental Health Continuum–Short Form, were randomly assigned to either the intervention group (*n* = 30) or a wait-list control group (*n* = 30). The intervention comprised eight weekly group sessions integrating guided music listening, visual creation, movement or role-play, and group sharing. Outcomes were assessed over time and analyzed for Group × Time interactions.

**Results:**

Significant Group × Time interactions were observed for flourishing, *F*(2, 112) = 4.57, *p* = 0.012, partial *η*^2^ = 0.075; positive affect *F*(2, 112) = 19.45, *p* < 0.001, partial *η*^2^ = 0.258; aesthetic experience *F*(2, 112) = 24.37, *p* < 0.001, partial *η*^2^ = 0.303; and the relaxation index *F*(2, 112) = 3.30, *p* = 0.040, partial *η*^2^ = 0.056. The Group × Time interaction for the attention index was not significant.

**Discussion:**

The findings provide preliminary evidence that a group-based multimodal art intervention may enhance aesthetic engagement, positive affect, psychological flourishing, and alpha-based relaxation among lonely university students, although attention-related EEG indices remained unchanged. Multimodal art programs may offer a feasible and accessible approach to supporting university students’ well-being.

## Introduction

University students have been increasingly recognized as a population at elevated risk for loneliness and diminished psychological well-being. Recent large-scale reviews indicate that depressive and anxiety symptoms remain highly prevalent in undergraduate populations, while post-pandemic evidence further suggests that loneliness is closely intertwined with distress and impaired daily functioning (Ahmed et al., 2023; Akram et al., 2025; Hunsmann et al., 2025; Li et al., 2022; Paiva et al., 2025; Wodika et al., 2025; World Health Organization, 2025). The public health relevance of loneliness extends beyond subjective discomfort: it has been linked to poorer mental health and elevated mortality risk, prompting international health agencies to reframe social connection as a core determinant of health rather than as a peripheral psychosocial concern (Holt-Lunstad et al., 2015; Reinhardt et al., 2021; World Health Organization, 2025). This issue is particularly salient for university students because the transition into higher education typically disrupts existing social ties at a time when academic demands and identity exploration intensify, leaving students more vulnerable to social disconnection and low positive functioning (Qirtas et al., 2023; Xie et al., 2025).

Although much research on student mental health has focused on symptomatic outcomes such as depression and anxiety, a prevention-oriented perspective also requires attention to positive mental health. Psychological flourishing has been theorized as optimal functioning across domains of meaning, competence, and supportive relationships, and is widely regarded as more than the mere absence of psychological symptoms (Diener et al., 2010; Keyes, 2002; Ryff, 1989; Seligman, 2011). Keyes’ mental health continuum, for instance, distinguishes flourishing from languishing and emphasizes that individuals may exhibit low positive functioning even when they do not meet criteria for a clinical disorder (Keyes, 2002, 2005). The Flourishing Scale, developed as a brief self-report measure of psychosocial prosperity, captures perceived success across important life domains including relationships and purpose (Diener et al., 2010). The construct is therefore especially relevant for lonely students, in whom loneliness may erode not only affective mood but also the relational and meaning-related dimensions of positive functioning (Cacioppo and Cacioppo, 2018; Fredrickson, 2001; Lyubomirsky et al., 2005).

Creative arts interventions have emerged as a promising approach for supporting mental health across clinical, educational, and community settings. A major scoping review commissioned by the WHO synthesized evidence from more than 3,000 studies and concluded that arts engagement may contribute to both prevention and treatment across the lifespan (Fancourt and Finn, 2019). Subsequent reviews have suggested that music engagement, visual art making, and other forms of creative expression can reduce adverse psychological outcomes, although the quality and consistency of evidence vary across populations and intervention formats (Trupp et al., 2025; Uttley et al., 2015). Active visual art therapy, music-based interventions, and group-based creative arts therapy can improve psychosocial well-being and alleviate depressive or anxious symptoms among college and community samples; at the same time, this literature continues to be characterized by substantial heterogeneity and an ongoing need for more rigorous randomized designs (Jensen et al., 2024; Joschko et al., 2024; Lin and Li, 2025; Quinn et al., 2025; Wang et al., 2025; Zhang et al., 2025).

Multimodal art intervention is theoretically well suited to lonely university students for several reasons. Art making provides a non-verbal and symbolic route for emotional expression, which may be particularly useful when individuals struggle to articulate distress directly (Hu et al., 2021; Van Lith, 2016). Within a single program, music, visual creation, movement, and group sharing engage complementary affective and interpersonal processes, offering repeated occasions for self-reflection and reconnection with others (Fancourt and Finn, 2019; Karkou et al., 2019; Koch et al., 2019). Aesthetic experience itself may also constitute an active ingredient of arts-based change. Aesthetic emotions such as being moved or fascinated are not merely pleasant by-products but may shape attention and openness to meaning-making during creative engagement, with recent neurocognitive accounts proposing that such relational aesthetic engagement is central to how creative arts therapies operate (Castellotti et al., 2025; Menninghaus et al., 2019; Schindler et al., 2017; Vaisvaser et al., 2024). Consistent with these considerations, brief creative arts therapy workshops have been associated with gains in perceived well-being and reductions in loneliness in university samples, and performing arts programs have shown comparable promise for improving social connection and creative expression (Agres and Chen, 2025; Arriaga et al., 2024).

Despite this growing evidence base, important gaps remain. Many arts-based studies focus on clinical symptoms or single-modality interventions, and relatively few randomized controlled trials have examined whether a structured multimodal art program can improve flourishing-related outcomes among nonclinical but psychologically vulnerable students (Joschko et al., 2024; Xue and Syed Abdullah, 2025). Existing trials also tend to emphasize immediate pre-post outcomes, leaving follow-up effects less well characterized (Jensen et al., 2024; Sayer et al., 2024). A further limitation concerns mechanism measurement. Although aesthetic experience is central to arts interventions, it is rarely assessed as an explicit outcome alongside flourishing and affective change (Schindler et al., 2017). Group arts interventions are similarly assumed to operate through relaxation or embodied regulation, but physiological indicators are seldom included, and they should be interpreted cautiously when consumer-grade wearable EEG systems are used (Cannard et al., 2021; Mikhaylov et al., 2024; Sugimoto et al., 2026).

Resting-state EEG offers one possible exploratory window into physiological change following arts-based intervention. Alpha-band activity has frequently been linked to relaxation and internally oriented states, although the direction and interpretation of alpha effects depend on the task context and analytic decisions involved (Lomas et al., 2015; Sugimoto et al., 2026). With respect to attention, the theta/beta ratio has been discussed as a marker of attentional control, with higher values often interpreted as reduced attentional engagement (Angelidis et al., 2018; Putman et al., 2014; Wang et al., 2024). The inverse, beta/theta, has been adopted in recent arousal- and engagement-oriented EEG work and in EEG-based art intervention research to index relatively greater attentional activation, with higher values reflecting stronger beta predominance over theta (Chen and Zeng, 2025; Wu et al., 2025).

The present study addressed these gaps by testing an eight-week group-based multimodal art intervention for lonely university students using a waitlist-controlled design with assessments at baseline, post-intervention, and three-month follow-up. The aim was to examine whether the intervention improved trait flourishing, state positive affect, aesthetic experience, and exploratory wearable EEG indices of relaxation and attention relative to a waitlist control. Flourishing and positive affect were examined as the primary and key secondary outcomes, whereas aesthetic-emotion and EEG indices were examined on an exploratory basis. Participants were assigned to a multimodal art intervention or waitlist group, completed self-report measures and five-minute resting EEG recordings at three time points, and outcomes were analyzed using a 2 × 3 mixed design. The study contributes preliminary controlled evidence that multimodal art intervention may support flourishing-related psychological functioning in lonely university students while clarifying that aesthetic and relaxation-related outcomes appear more responsive to short-term intervention than attention-related EEG indices.

## Methods

### Participants and group assignment

Participants were undergraduate or postgraduate university students aged 18 to 22 years. Eligibility required elevated loneliness, defined as a UCLA Loneliness Scale-20 score of 44 or above, and a Mental Health Continuum-Short Form (MHC-SF) classification of languishing or moderate mental health, determined using the categorical diagnostic algorithm of Keyes (2002, 2005). Exclusion criteria included current psychological treatment, a history of severe mental disorder, and current suicide risk. Current suicide risk was screened using item 9 of the PHQ-9 together with a brief screening interview. Students who endorsed active suicidal ideation were not enrolled and were referred to the university counseling center. Recruitment and screening occurred between November 2025 and January 2026. Of 83 students screened, 60 met eligibility criteria and consented to participate. The remaining 23 screened students were not randomized because 18 declined participation for practical reasons and 5 declined because of preferences regarding group allocation. Eligible consenting participants were assigned at a 1:1 ratio to either the intervention group (*n* = 30) or the waitlist control group (*n* = 30) using block randomization. Two intervention-group participants withdrew after baseline because of time constraints, leaving 28 intervention participants and 30 waitlist participants with complete post-intervention and follow-up data. Written informed consent was obtained from all participants before enrollment. Ethical approval was granted by the institutional review board of Jinzhou Medical University [JZMULL2026334]. A CONSORT participant flow diagram is provided in Figure 1 (Moher et al., 2010; Schulz et al., 2010).

**Figure 1:**
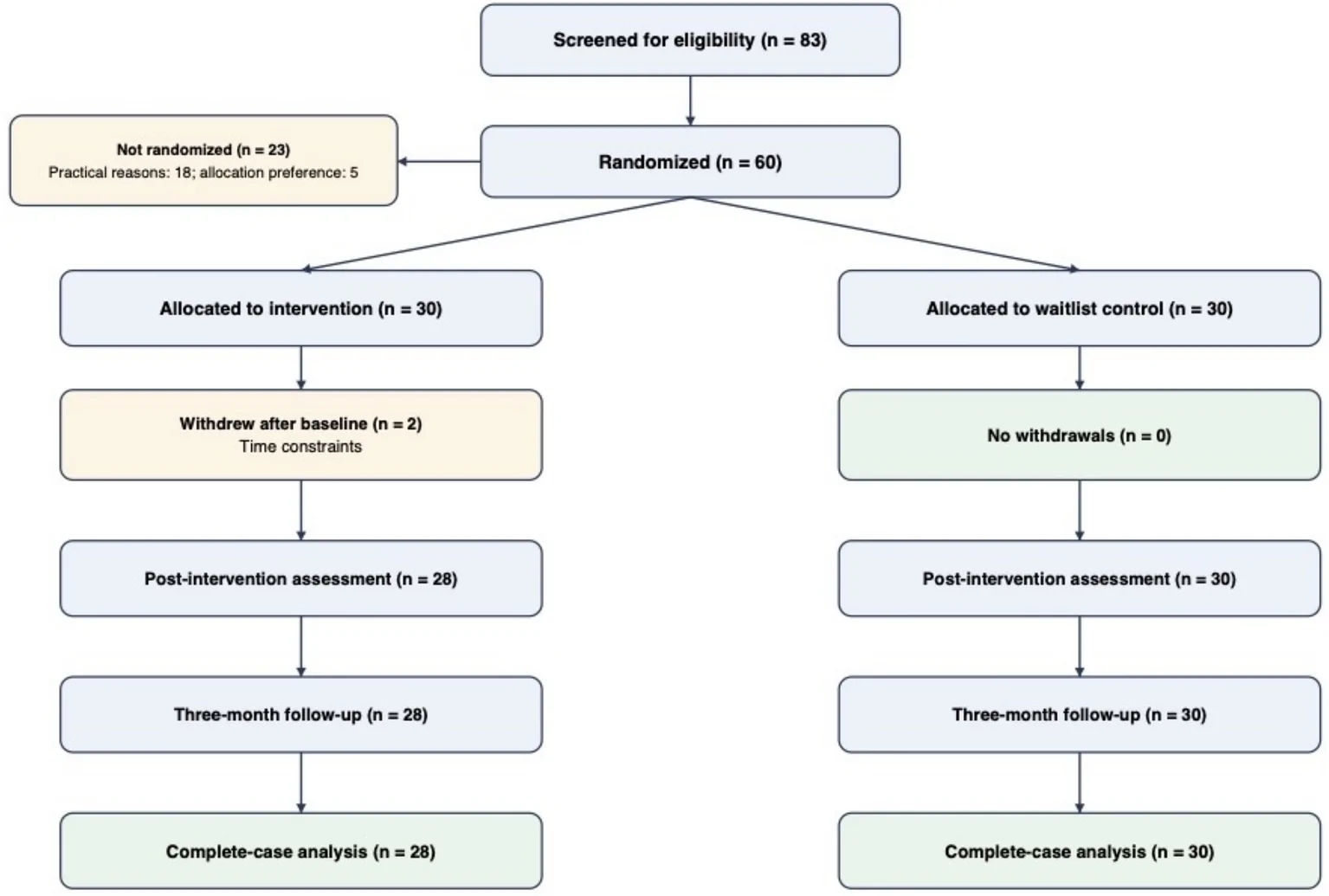
Consort flow.

### Intervention

The intervention consisted of eight weekly group sessions, each lasting approximately 90 to 120 min. Three parallel intervention groups were conducted, with 8 to 10 participants per group. All sessions were delivered by the same trained facilitator (the first author), who had completed 40 h of structured group arts facilitation training and received regular supervision from a licensed art therapist during the study period. A standardized session manual specified a consistent sequence of opening and orientation, guided music listening, visual creative response to music, movement or role-play activity, group sharing, and closing reflection. The eight weekly themes progressed from self-awareness and emotional dialogue, through expression of difficulties, identity exploration, and connection with others, to identification of personal strengths, hope mapping, and integration. Intervention delivery was monitored against the standardized manual; no protocol deviations or adverse events were reported. Participants assigned to the waitlist condition did not receive any art-based intervention between T0 and T2. To preserve ethical acceptability, the waitlist group was offered the same intervention after completion of the T2 follow-up assessment.

### Measures

Flourishing. Trait flourishing was assessed using the eight-item Diener Flourishing Scale (Diener et al., 2010). We used the Simplified Chinese version validated by Tang et al. (2016). Items were rated on a 7-point Likert scale from 1 to 7 and were summed such that higher scores indicate higher flourishing.

Positive affect. State positive affect was assessed using the positive affect subscale of the Positive and Negative Affect Schedule (PANAS-PA; Watson et al., 1988). A Chinese version was used (Bao et al., 2020). The 10 positive affect items were rated on a 5-point scale from 1 to 5 and summed, with higher scores indicating stronger positive affect.

Aesthetic emotions. Aesthetic emotions were assessed using a 21-item selection based on the Aesthetic Emotions Scale (AESTHEMOS; Schindler et al., 2017). The original AESTHEMOS comprises 21 two-item subscales; in the present study, one item representing each emotion domain was administered on a 5-point response scale. Items 16–20 (ugliness, boredom, confusion, anger, and uneasiness) were reverse-scored using 6 − *x*, where *x* represents the original item score; the sadness item was retained in its original direction. The resulting aggregate score was used as an exploratory study-specific indicator of overall positively valenced aesthetic response, with higher values indicating a more positive overall response profile. Because aesthetic emotions are multidimensional and this aggregation was not intended to establish a unidimensional validated total score, this outcome was interpreted cautiously and as exploratory. At each assessment, participants rated their aesthetic-emotion experiences in relation to the art-related activities specified in the assessment instructions. All study measures were reviewed and pilot-tested by the research team before administration.

Baseline covariates and screening measures. Loneliness was screened using the Chinese UCLA Loneliness Scale-20 (Russell, 1996; Ip et al., 2024). A score of 44 or higher was used as an operational eligibility threshold for relatively elevated loneliness; it was not treated as a diagnostic cutoff. Baseline depressive symptoms, anxiety symptoms, and openness to experience were assessed using the PHQ-9 (Kroenke et al., 2001), GAD-7 (Spitzer et al., 2006), and the BFI-2 Openness scale (Soto and John, 2017; Zhang et al., 2022), respectively. PHQ-9 and GAD-7 items were scored from 0 to 3, and BFI-2 Openness items from 1 to 5. The PHQ-9 and GAD-7 were administered in their validated Chinese versions (He et al., 2010; Wang et al., 2014).

### EEG acquisition and calculation of EEG indices

Resting-state EEG was recorded for 5 min with eyes closed at T0, T1, and T2 using a BrainLink Pro wearable EEG system (Macrotellect Ltd., Shenzhen, China). The device recorded resting frontal EEG. Band-amplitude values were derived from the raw recordings through the manufacturer’s processing pipeline and were obtained directly from the device provider (Macrotellect Ltd.). Participants were seated comfortably and instructed to keep their eyes closed, remain still, and minimize blinking and other movements during recording. The theta (4–7 Hz), alpha (8–13 Hz), and beta (13–30 Hz) band-amplitude values were labeled theta_uV, alpha_uV, and beta_uV. Two ratio-based variables were then calculated from the exported band-amplitude values and averaged across each five-minute recording: alpha/beta (*α*/*β*) and beta/theta (*β*/*θ*). These wearable EEG-derived ratios were treated as exploratory physiological measures. They were not interpreted as direct measures of relaxation or attentional functioning, nor as clinical or diagnostic indicators.


$$\text{Relaxation index}=\frac{\text{alpha}}{\text{beta}}$$

Attention index=beta/theta

A higher relaxation index reflects stronger alpha activity relative to beta activity, indexing a more relaxed resting state. A higher attention index reflects stronger beta activity relative to theta activity, indexing relatively greater attentional engagement. These EEG variables were treated as exploratory physiological outcomes rather than as clinical diagnostic indicators.

### Data analysis

Descriptive statistics were reported as means and standard deviations for continuous variables and as frequencies and percentages for categorical variables. Baseline characteristics were reported descriptively by randomized group; baseline significance tests were not interpreted as evidence of successful randomization. Internal consistency was evaluated with Cronbach’s alpha (Cronbach, 1951). For the primary analyses, each outcome was analyzed using a mixed analysis of variance (ANOVA) with Group as the between-subjects factor and Time as the within-subjects factor; the Group × Time interaction was the effect of primary interest. Psychological flourishing was treated as the primary outcome, positive affect as a key secondary outcome, and aesthetic-emotion and wearable EEG measures as exploratory outcomes. This outcome hierarchy was specified for transparent interpretation during revision and was not prospectively registered. Partial eta squared was reported as the effect size. Where appropriate, simple between-group effects at each time point were summarized using Cohen’s d. All tests were two-sided with alpha set at 0.05. The target sample size of 60 was based on recruitment feasibility rather than an *a priori* power analysis. Because two intervention-group participants withdrew after baseline, the mixed ANOVA models were estimated using the complete-case sample of 58 participants. Descriptive statistics at each time point are based on all available observations, with relevant sample sizes shown in Table 1. The lack of prospective trial registration and the use of complete-case analysis are acknowledged as limitations.

**Table 1:** Descriptive statistics by group and time point.

Outcome	Group	T0 M ± SD (n)	T1 M ± SD (*n*)	T2 M ± SD (*n*)
Flourishing	Intervention	32.43 ± 5.51 (30)	36.18 ± 6.58 (28)	34.46 ± 6.75 (28)
Flourishing	Waitlist	33.53 ± 4.90 (30)	34.40 ± 5.97 (30)	34.07 ± 4.98 (30)
PANAS-PA	Intervention	25.87 ± 5.48 (30)	34.43 ± 4.98 (28)	31.04 ± 5.96 (28)
PANAS-PA	Waitlist	27.27 ± 7.53 (30)	29.33 ± 7.02 (30)	26.60 ± 6.86 (30)
Aesthetic experience	Intervention	57.70 ± 12.96 (30)	75.82 ± 11.04 (28)	72.00 ± 13.00 (28)
Aesthetic experience	Waitlist	61.50 ± 12.64 (30)	63.93 ± 11.26 (30)	63.33 ± 12.42 (30)
Relaxation index (*α*/*β*)	Intervention	1.362 ± 0.295 (30)	1.504 ± 0.369 (28)	1.425 ± 0.286 (28)
Relaxation index (*α*/*β*)	Waitlist	1.377 ± 0.298 (30)	1.398 ± 0.246 (30)	1.349 ± 0.283 (30)
Attention index (*β*/*θ*)	Intervention	1.032 ± 0.219 (30)	1.015 ± 0.252 (28)	1.014 ± 0.219 (28)
Attention index (*β*/*θ*)	Waitlist	0.987 ± 0.196 (30)	1.006 ± 0.242 (30)	1.011 ± 0.198 (30)

M = mean; SD = standard deviation; T0 = baseline; T1 = post-intervention; T2 = three-month follow-up. Descriptive statistics are based on all available observations at each time point; sample sizes are shown in parentheses. PANAS-PA = positive affect subscale of the positive and negative affect schedule. The aesthetic-emotion aggregate was computed after reverse-scoring items 16–20 and is interpreted as exploratory.

## Results

### Participant flow and baseline characteristics

Of the 83 students screened for eligibility between November 2025 and January 2026, 60 eligible students consented to participate and were randomized to the intervention group (*n* = 30) or the waitlist control group (*n* = 30). Eighteen screened students declined participation for practical reasons, and five declined because of preferences regarding group allocation. Two participants in the intervention group withdrew after baseline because of time constraints, yielding 58 participants with complete post-intervention and follow-up data. A CONSORT-style participant flow diagram is presented in Figure 1. Baseline characteristics are presented descriptively in Table 2; they should not be interpreted as formal tests of randomization success.

**Table 2:** Baseline characteristics by group.

Variable	Intervention (*n* = 30)	Waitlist (*n* = 30)	*p*
Age (years)	20.00 ± 1.11	20.20 ± 1.27	0.519
Art training (years)	0.53 ± 1.01	0.27 ± 0.52	0.205
UCLA loneliness	48.67 ± 2.70	48.37 ± 2.95	0.683
PHQ-9	10.90 ± 5.93	8.90 ± 5.73	0.189
GAD-7	8.63 ± 5.54	8.80 ± 5.13	0.904
BFI-2 openness	41.47 ± 6.97	43.03 ± 6.22	0.362
Female, *n* (%)	18 (60.0)	17 (56.7)	0.793
Languishing on MHC-SF, *n* (%)	15 (50.0)	17 (56.7)	0.605

Values are M ± SD unless otherwise stated. UCLA = UCLA Loneliness Scale-20; PHQ-9 = Patient Health Questionnaire-9; GAD-7 = Generalized Anxiety Disorder-7; BFI-2 = Big Five Inventory-2; MHC-SF = Mental Health Continuum-Short Form.

### Scale reliability

Internal consistency was acceptable to good across all self-report measures. Cronbach’s alpha values at baseline were 0.829 for the PHQ-9, 0.827 for the GAD-7, and 0.777 for the BFI-2 Openness scale. The Diener Flourishing Scale showed good reliability across T0, T1, and T2 (*α* = 0.829, 0.830, and 0.830, respectively). PANAS-PA also showed good reliability across the three time points (*α* = 0.846, 0.850, and 0.851), as did the aesthetic-emotion aggregate (*α* = 0.880, 0.882, and 0.880).

### Descriptive statistics

Descriptive statistics for the five outcomes across group and time are presented in Table 1. Values at each time point are based on all available observations; therefore, the intervention group contributes data from 30 participants at T0 and 28 participants at T1 and T2. From T0 to T1, the intervention group showed increases in flourishing, PANAS-PA, the exploratory aesthetic-emotion aggregate, and the *α*/*β* ratio, with partial attenuation at T2. The waitlist group showed smaller changes over time, and the *β*/*θ* ratio remained relatively stable in both groups.

### Primary mixed ANOVA results

The main complete-case mixed ANOVA results are summarized in Table 3. The Group × Time interaction was significant for flourishing, *F*(2, 112) = 4.57, *p* = 0.012, partial *η*^2^ = 0.075. For positive affect, the Group × Time interaction was also significant, *F*(2, 112) = 19.45, *p* < 0.001, partial *η*^2^ = 0.258. A significant Group × Time interaction was likewise observed for the exploratory aesthetic-emotion aggregate, *F*(2, 112) = 24.37, *p* < 0.001, partial *η*^2^ = 0.303. For the exploratory wearable EEG-derived ratios, the Group × Time interaction was nominally significant for *α*/*β*, *F*(2, 112) = 3.30, *p* = 0.040, partial *η*^2^ = 0.056, whereas the interaction for *β*/*θ* did not reach significance, *F*(2, 112) = 0.96, *p* = 0.385, partial *η*^2^ = 0.017. Given the exploratory status of the EEG outcomes and the multiple outcomes examined, the *α*/*β* finding is interpreted cautiously. The overall pattern of group-by-time change is shown in Figure 2.

**Table 3:** Group × time interaction results from the mixed ANOVA models.

Outcome	*F*	*df*	*p*	Partial *η*^2^	Result
Flourishing	4.57	2, 112	0.012	0.075	Significant
PANAS-PA	19.45	2, 112	<0.001	0.258	Significant
Aesthetic experience	24.37	2, 112	<0.001	0.303	Significant
Relaxation index (*α*/*β*)	3.30	2, 112	0.040	0.056	Significant
Attention index (*β*/*θ*)	0.96	2, 112	0.385	0.017	N.S.

N.S. = not significant. Results are from 2 (Group) × 3 (Time) complete-case mixed ANOVAs estimated on *N* = 58 participants. Flourishing was the primary outcome; positive affect was a key secondary outcome; aesthetic-emotion and wearable EEG outcomes were exploratory.

**Figure 2:**
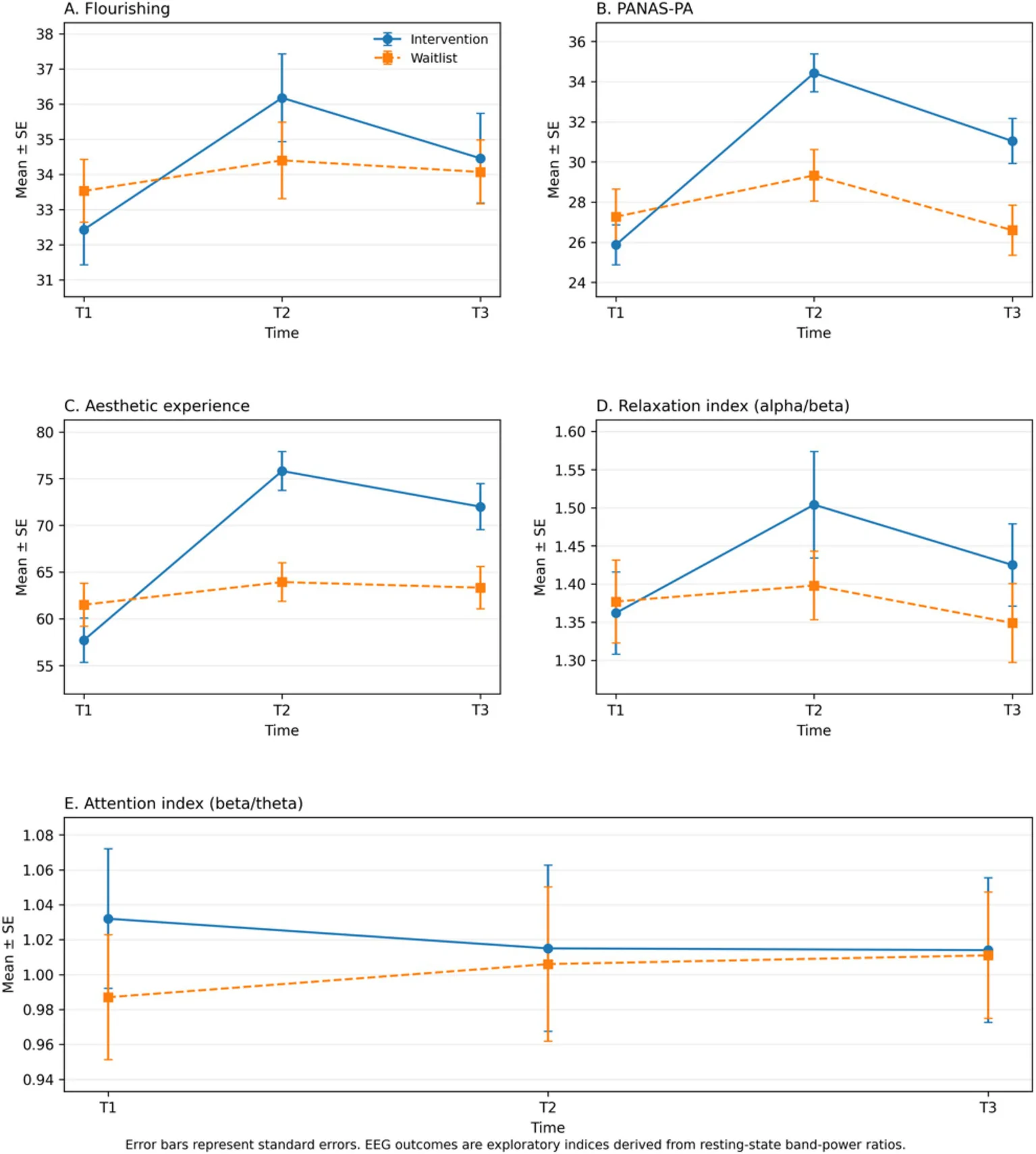
Changes in study outcomes across the three assessment time points in the intervention and wait-list groups. **(A)** Psychological flourishing; **(B)** positive affect, assessed using the Positive Affect subscale of the Positive and Negative Affect Schedule (PANAS-PA); **(C)** aesthetic experience; **(D)** EEG-derived relaxation index (alpha/beta ratio); and **(E)** EEG-derived attention index (beta/theta ratio). Data are presented as means ± standard errors. The EEG outcomes were exploratory indices derived from resting-state frequency-band power ratios.

To characterize the between-group simple effects underlying the interactions, Cohen’s d (intervention vs. waitlist, pooled SD) was computed at each time point. Baseline (T0) between-group effects were small for all outcomes (|d| ≤ 0.30). At post-intervention (T1), effect sizes favored the intervention group and were largest for the exploratory aesthetic-emotion aggregate (d = 1.07, 95% CI [0.52, 1.62]) and positive affect (d = 0.83, 95% CI [0.29, 1.37]), small-to-moderate for the *α*/*β* ratio (d = 0.34, 95% CI [−0.18, 0.86]) and flourishing (d = 0.28, 95% CI [−0.24, 0.80]), and negligible for the *β*/*θ* ratio (d = 0.04, 95% CI [−0.48, 0.56]). At the three-month follow-up (T2), effects were attenuated but remained moderate descriptively for positive affect (d = 0.69, 95% CI [0.16, 1.22]) and the exploratory aesthetic-emotion aggregate (d = 0.68, 95% CI [0.15, 1.21]). These descriptive effect sizes do not by themselves establish maintenance of effects; follow-up conclusions are therefore stated cautiously.

## Discussion

This study examined the effects of an eight-week multimodal art intervention on psychological flourishing, state positive affect, aesthetic emotions, and exploratory wearable EEG-derived ratios among lonely university students. Relative to the waitlist group, the intervention group showed differential change over time in flourishing, positive affect, the exploratory aesthetic-emotion aggregate, and the *α*/*β* ratio, whereas the *β*/*θ* ratio did not show a significant Group × Time interaction. The observed pattern is consistent with, but did not prospectively test, a distinction between more state-sensitive outcomes and broader psychosocial functioning. Given the modest sample, lack of prospective registration, use of a waitlist control, and exploratory outcome analyses, the findings should be regarded as preliminary.

The significant but modest improvement in flourishing is theoretically meaningful. Flourishing reflects relatively broad psychosocial functioning, including perceived competence, purpose, and the quality of one’s relationships (Diener et al., 2010; Keyes, 2002). Because these dimensions are partly trait-like and embedded in students’ ongoing social and academic lives, large short-term changes should not necessarily be expected. The present pattern—flourishing improved after intervention but with a smaller effect than positive affect and aesthetic experience—fits the view that positive functioning may change gradually through repeated experiences of meaning, mastery, and interpersonal affirmation (Fredrickson, 2001; Lyubomirsky et al., 2005; Ryff, 1989; Seligman, 2011). For lonely students, even modest gains in flourishing may be important, because low positive functioning can persist in the absence of severe clinical symptoms (Keyes, 2005; World Health Organization, 2025).

State positive affect showed a stronger intervention response than trait flourishing. This finding is consistent with the view that affective outcomes are more sensitive to brief and medium-term psychosocial interventions than broad trait-level well-being constructs (Watson et al., 1988). The multimodal art sessions included guided music listening, visual creation, movement or role play, and group sharing, all of which may provide immediate opportunities for enjoyment, emotional release, and shared meaning. Positive affect may therefore function as an early and responsive indicator of intervention benefit, whereas flourishing may require longer intervention exposure or broader environmental support to consolidate (Fredrickson, 2001; Lyubomirsky et al., 2005). This interpretation is compatible with recent evidence that arts-based and well-being interventions often produce measurable effects on affective and social well-being even when broader flourishing effects are smaller or more variable (Agres and Chen, 2025; Arriaga et al., 2024; Jensen et al., 2024).

The largest observed group-by-time difference involved the exploratory aesthetic-emotion aggregate. This outcome should be interpreted cautiously. The AESTHEMOS framework captures multiple aesthetic-emotion domains, including positive, activating, calming, and negatively valenced responses (Schindler et al., 2017). In the present study, selected items were aggregated after reverse scoring of several negatively valenced items to create a study-specific indicator of overall positively valenced aesthetic response. This score should not be treated as a validated unidimensional measure or as evidence of an intervention mechanism. Rather, the finding suggests that future arts-intervention studies should use theoretically specified and psychometrically validated measures of aesthetic engagement and test proposed mechanisms with appropriately designed mediation analyses.

The exploratory EEG findings require a cautious interpretation. The wearable EEG-derived *α*/*β* ratio showed a nominally significant Group × Time interaction, whereas the *β*/*θ* ratio did not. These ratios were calculated from frontal wearable EEG band-amplitude values and should not be interpreted as direct measures of relaxation or attentional functioning. The use of a consumer-grade wearable EEG system and multiple outcome testing further limit inference; thus, the *α*/*β* result should be regarded as exploratory physiological evidence rather than evidence of a neural mechanism. The null finding for *β*/*θ* is also informative, but it should likewise not be interpreted as a definitive test of attentional change.

These findings extend previous research in several ways. Earlier reviews have shown that art therapy, dance movement therapy, music-related interventions, and arts-on-prescription programs can improve psychological outcomes, although the evidence base remains heterogeneous across design quality, population, and outcome selection (Hu et al., 2021; Jensen et al., 2024; Joschko et al., 2024; Karkou et al., 2019; Quinn et al., 2025; Uttley et al., 2015; Zhang et al., 2025). The present study focuses on lonely university students rather than a diagnosed clinical sample, targets positive functioning in addition to symptoms, and includes exploratory aesthetic-emotion and physiological indicators. The program may be an accessible group-based option for supporting student well-being; however, stigma was not directly measured and no conclusion about a low-stigma effect can be drawn.

At the same time, the waitlist-controlled design limits causal specificity. Although the study supports internal validity for the comparison between intervention and no immediate intervention, the design cannot separate multimodal art-specific effects from nonspecific effects such as expectancy, facilitator attention, or simply attending a structured weekly group. This limitation is particularly relevant because group sharing and interpersonal presence are integral to the intervention and may themselves reduce loneliness or enhance well-being (Holt-Lunstad et al., 2015; World Health Organization, 2025). Future studies should consider active control conditions—such as health education, non-art group discussion, or structured leisure activities—to determine whether the benefits are specific to aesthetic and expressive processes or instead reflect broader group-support mechanisms. Reporting should also continue to include transparent documentation of group allocation, attrition, missing data handling, and the specification of primary and secondary outcomes.

Several additional limitations should be noted. First, the sample size was modest, and the findings should be interpreted as preliminary rather than definitive evidence. Second, the study was not prospectively registered; accordingly, the outcome hierarchy was clarified during revision and the findings require replication in preregistered trials. Third, the primary analyses used complete cases because two intervention participants withdrew after baseline; this approach may be sensitive to assumptions about missingness. Because only two of 60 participants (3.3%) withdrew, complete-case and intention-to-treat estimates are unlikely to differ substantially. Fourth, the waitlist-controlled design cannot separate art-specific effects from expectancy, facilitator attention, or participation in a structured weekly group. In addition, all sessions were delivered by a single facilitator to stable intervention cohorts, so facilitator and cohort effects cannot be separated from the intervention itself. Fifth, the primary psychological outcomes relied on self-report and are vulnerable to expectancy and social-desirability influences. Sixth, the three-month follow-up provides information about short-term follow-up patterns but does not by itself establish long-term durability. Finally, the wearable EEG device provided exploratory frontal band-amplitude metrics rather than a research-grade, high-density EEG assessment; the *α*/*β* and *β*/*θ* ratios should not be overinterpreted as direct neural mechanisms.

Several directions for future research follow from these findings. Larger preregistered trials with active control groups are needed to estimate intervention-specific effects more precisely. Future studies could also measure loneliness, social connectedness, and group cohesion alongside flourishing, to clarify whether relational change is one of the pathways through which multimodal art intervention benefits lonely students. More intensive measurement of aesthetic experience after each session could help identify which components of the intervention produce the strongest emotional and interpersonal engagement. Finally, studies using research-grade EEG, heart-rate variability, or other multimodal psychophysiological assessment could examine whether relaxation-related physiological changes mediate improvements in affect or well-being. Such work would help move the field from evidence that arts interventions are beneficial toward clearer specification of when, for whom, and through which processes they work (Fancourt and Finn, 2019; Jensen et al., 2024; Kazdin, 2007).

In conclusion, this exploratory randomized waitlist-controlled study provides preliminary evidence consistent with potential benefits of an eight-week multimodal art intervention for psychological flourishing and positive affect among lonely university students. The largest observed change involved an exploratory study-specific aesthetic-emotion aggregate, while the wearable EEG-derived *α*/*β* finding should be interpreted cautiously and the *β*/*θ* ratio was unchanged. Future preregistered trials with larger samples, active control conditions, validated aesthetic-emotion scoring, and research-grade physiological measures are needed to determine the specificity, durability, and mechanisms of these effects.

## Data Availability

The raw data supporting the conclusions of this article will be made available by the authors, without undue reservation.
